# Early detection strategies to prevent Rapunzel syndrome in low-resource settings

**DOI:** 10.1097/RC9.0000000000000508

**Published:** 2026-05-06

**Authors:** Gamini Goonetilleke, Ruwan Jayatunge, Henri Lenoir

**Affiliations:** a Sri Jayawardanapura General Hospital, Nugegoda, Sri Lanka; b Edith Cowan University, Colombo, Sri Lanka; c Uniformed University of the Health Sciences, Bethesda, Maryland, USA

Trichobezoars are accumulations of ingested hair within the gastrointestinal tract, most often arising in individuals with trichotillomania and trichophagia[1]. When the mass extends beyond the pylorus into the small intestine, it is termed Rapunzel syndrome – a rare but serious consequence of chronic hair ingestion^[^2,3^]^. Although uncommon, trichobezoars, and even more so Rapunzel syndrome, represent a late physical manifestation of an underlying psychiatric disorder.

In resource-limited settings, patients with trichotillomania frequently present late, often after months of nonspecific gastrointestinal symptoms such as pain, vomiting, or weight loss. Limited access to early psychiatric evaluation allows progression to gastric outlet obstruction, intestinal perforation, or peritonitis, often requiring open surgical intervention^[^4–6^]^. Early identification and psychiatric treatment of trichotillomania are therefore essential to prevent this potentially fatal yet preventable surgical emergency.

Here, we report the case of a 14-year-old female patient from a remote district in Sri Lanka who presented with a 3-month history of upper abdominal pain, recurrent vomiting, anorexia, and progressive weight loss. Multiple prior consultations at district and provincial hospitals, including an abdominal CT scan, had failed to establish a diagnosis. On admission to Sri Jayawardenepura General Hospital, the patient was pale, wasted, and dehydrated. Abdominal examination revealed no palpable mass. Endoscopy showed an obstruction at the gastric inlet with visible strands of hair. Exploratory laparotomy demonstrated a markedly distended stomach filled with a firm, matted intragastric mass extending into the duodenum and jejunum, confirming Rapunzel syndrome. A large trichobezoar was removed through an anterior gastrotomy. The remainder of the bowel was palpated to ensure clearance of the trichobezoar, and the anterior gastrotomy was closed in two layers. The patient subsequently recovered well and underwent psychiatric evaluation and follow-up. Written informed consent was obtained from the patient for publication.

Although Rapunzel syndrome remains exceedingly rare, reported cases in the literature are primarily from low- and middle-income countries (LMICs). A review of recent literature identified individual case reports from Nepal, India, Tunisia, and the United Arab Emirates, as well as isolated reports from Italy and Ireland[7] (Fig. 1). The predominance of cases from LMICs likely reflects limited access to psychiatric care and delayed diagnosis rather than a truly higher incidence.

**Figure 1.**
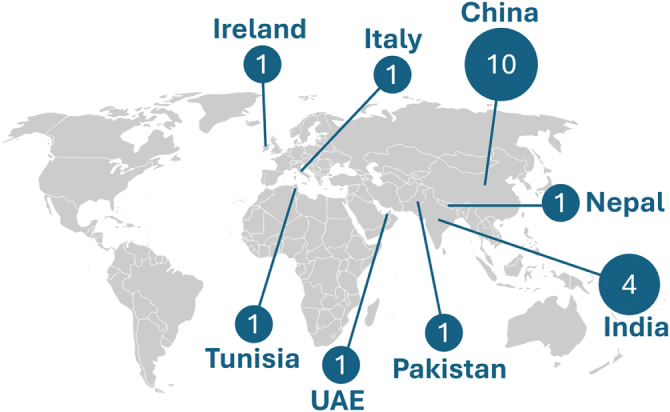
Global distribution of Rapunzel syndrome cases based on the review by Iqbal *et al*[7].

There is a large gap in mental health service availability between LMICs and high-income countries. In addition, mental health resources are concentrated in more urban environments, where tertiary hospitals and mental hospitals are centralized[8]. As a result, much of the burden in mental health diagnosis and management falls onto primary care providers. These providers should be trained to utilize low-cost imaging modalities to aid in their differential rather than providing tailored training on how to recognize diagnoses such as trichotillomania and subsequent complications of trichobezoar formation. In addition, the clinical features of trichobezoar and Rapunzel syndrome are already fairly nonspecific – vague epigastric pain, nausea, vomiting, bloating, and weight loss. As a result, diagnosis is often delayed until complications arise. In resource-constrained environments, simple, low-cost imaging such as ultrasound could play a critical role in early detection. Identifying a phytobezoar on ultrasound in a patient with suggestive symptoms could prompt further evaluation and early referral for endoscopy, potentially avoiding laparotomy and gastrotomy. The use of ultrasound detection with a curvilinear probe has been described in the literature: place the patient in a supine position, identify the stomach, and evaluate the intra-gastric mass in two orthogonal views to visualize a bright, hyperechoic curvilinear line with strong posterior acoustic shadowing[9].

The case presented and similar ones discussed in this journal underscore the importance of early recognition of trichotillomania and accessible diagnostic evaluation in LMICs. Training non-specialist providers to identify behavioral clues, coupled with low-cost diagnostic pathways such as ultrasound triage, could substantially reduce the morbidity associated with Rapunzel syndrome. Where endoscopy is available, timely intervention could prevent progression to surgical emergencies, reduce hospital costs, and improve psychological recovery.

Ultimately, Rapunzel syndrome exemplifies the intersection of mental health and surgery. Preventing such cases requires not only operative expertise but also awareness and integrated mental health services. For many patients in resource-limited environments, these simple steps could transform a potentially fatal emergency into a preventable condition.

## Data Availability

Data are available by contacting the corresponding author.
